# A Single Exercise Session Elicits a Transient Kynurenine Metabolic Signature in Health and Disease: A Systematic Review and Meta‐Analysis

**DOI:** 10.1096/fj.202603422R

**Published:** 2026-10-13

**Authors:** Marcus Vinicius dos Santos Rangel, Gabriela Andrade Paz, Maria das Graças C. Souza, Marie Kupjetz, Tiffany Y. Wences Chirino, Philipp Zimmer, Paulo Farinatti, Juliana Pereira Borges

**Affiliations:** ^1^ Laboratory of Physical Activity and Health Promotion, Institute of Physical Education and Sports University of Rio de Janeiro State Rio de Janeiro Rio de Janeiro Brazil; ^2^ Clinical and Experimental Research Laboratory on Vascular Biology (BioVasc) University of Rio de Janeiro State Rio de Janeiro Rio de Janeiro Brazil; ^3^ Research Group “Sports Medicine”, Institute for Sport and Sport Science TU Dortmund University Dortmund Germany

**Keywords:** chronic disease, exercise, kynurenic acid, kynurenine, metabolism, tryptophan

## Abstract

Tryptophan (TRP) is primarily metabolized via the kynurenine (KYN) pathway, which plays a central role in energy homeostasis, as well as immune, inflammatory, and neurobiological regulation. Although a single exercise session may modulate this pathway, the magnitude and consistency of these responses remain unclear. Therefore, this meta‐analysis aimed to synthesize acute exercise–induced changes in KYN pathway metabolites. PubMed, Web of Science, and Scopus were searched from inception to June 2026. Clinical trials evaluating the effects of a single exercise session on circulating TRP and KYN pathway metabolites were included. Random‐effects meta‐analyses were performed using standardized mean differences (Hedges' g). Outcomes were analyzed separately for immediate post‐exercise responses (≤ 5 min) and recovery responses (> 5 min to 3 h). Prespecified subgroup analyses examined population characteristics, training status, and exercise modality. Of 3203 records, 24 clinical trials (*n* = 695) were included in the systematic review, and 20 provided data for meta‐analysis. Immediately after exercise, TRP decreased, whereas KYN, KYN/TRP, kynurenic acid (KYNA), and quinolinic acid (QA) increased. During recovery, reductions in TRP and elevations in KYN/TRP persisted, whereas KYN and QA returned toward baseline. KYNA remained elevated, while QA/KYNA remained unchanged. Subgroup analyses suggested larger responses in healthy individuals, trained participants, and following aerobic exercise, particularly for TRP and KYNA. Exercise induces heterogeneous changes in KYN metabolism, characterized by reduced TRP and increased KYN/TRP ratio and downstream metabolites. These responses are transient and context‐dependent, reflecting acute changes in metabolic flux rather than sustained pathway remodeling.

**Trial Registration:** PROSPERO, identifier CRD42022351481

## Graphical abstract

A single exercise session transiently modulates systemic kynurenine pathway metabolism. Immediately after exercise, tryptophan decreases while kynurenine, KYN/TRP, kynurenic acid, and quinolinic acid increase. During recovery, lower tryptophan, higher KYN/TRP, and kynurenic acid remain. In contrast, elevations in kynurenine and quinolinic acid do not remain, with kynurenine returning toward baseline and the quinolinic acid response attenuated. Responses are generally larger in healthy and trained participants and after aerobic exercise, supporting context‐dependent shifts in metabolic flux rather than sustained pathway remodeling.

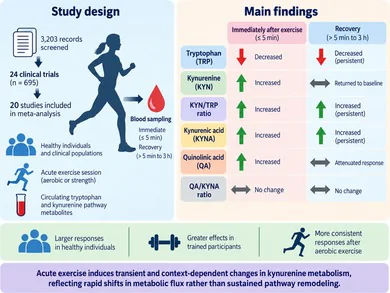

## 1 Introduction

Tryptophan (TRP) is an essential amino acid required for protein synthesis and a precursor for metabolic pathways, including the 5‐hydroxytryptamine, indole, and kynurenine (KYN) pathway. Approximately 95% of TRP degradation occurs via the KYN pathway, which serves as the only de novo route for nicotinamide adenine dinucleotide (NAD^+^) synthesis [1, 2]. Beyond its bioenergetic role, the KYN pathway generates metabolites involved in immune, metabolic, and neurobiological regulation, and its imbalance has been associated with cancer, chronic inflammatory, metabolic, cardiovascular, neurodegenerative, and psychiatric diseases [2, 3, 4, 5, 6].

Under non‐inflammatory conditions, TRP availability is primarily regulated by hepatic tryptophan‐2,3‐dioxygenase (TDO). Conversely, pro‐inflammatory cytokines, particularly interferon‐γ and tumor necrosis factor‐α, preferentially activate indoleamine‐2,3‐dioxygenase 1 (IDO1) in immune and peripheral tissues [7, 8, 9], favoring TRP degradation in extrahepatic tissues. KYN pathway metabolites may exert neurotoxic effects (e.g., quinolinic acid [QA]) [10] or neuroprotective actions (e.g., kynurenic acid [KYNA]) [11]. Accordingly, the QA/KYNA ratio has been proposed as an indirect marker of the balance between neurotoxic and neuroprotective pathway activity. The balance between these branches is critical for maintaining immune and neuronal homeostasis, positioning the KYN pathway as a promising therapeutic target [12, 13, 14].

Physiological stressors such as exercise may substantially influence the KYN pathway. By increasing energetic demands and challenging metabolic homeostasis, a single exercise session induces transient perturbations in metabolic, oxidative, endocrine, and immune activity [15, 16, 17, 18]. Exercise also increases circulating cytokines, particularly interleukin‐6, and modifies metabolic and hormonal conditions potentially involved in IDO1/TDO activation and downstream KYN metabolism [18, 19, 20]. Experimental evidence supports that repeated exercise exposure increases skeletal muscle expression of kynurenine aminotransferases (KATs), favoring the peripheral conversion of KYN into KYNA and reducing the availability of KYN for neurotoxic downstream metabolism [21, 22, 23].

However, whether a single exercise session consistently induces such favorable metabolic changes in humans remains unclear. While some studies reported increases in KYN and neurotoxic metabolites following strenuous exercise [24, 25, 26, 27, 28, 29, 30, 31], others observed enhanced KYNA production and anti‐inflammatory responses [19, 30, 32, 33, 34]. These discrepancies may reflect differences in exercise protocols in terms of modality, intensity, and/or duration, sampling time, training status, and participant characteristics. Individuals with chronic diseases may exhibit altered basal KYN pathway metabolism and inflammatory activation, potentially leading to responses distinct from those observed in healthy individuals [15].

Despite growing interest in exercise‐induced modulation of the KYN pathway, previous reviews have primarily emphasized mechanistic interpretations, muscle–brain signaling, or disease‐specific perspectives [17, 24, 35, 36]. A comprehensive quantitative synthesis examining whether a single exercise session systematically modulates circulating KYN pathway metabolites, and to what extent these responses are influenced by exercise‐related and population‐specific characteristics, is still lacking.

Therefore, this systematic review and meta‐analysis tested the hypothesis that exercise modulates KYN pathway metabolism in humans, favoring a transient shift toward increased production of protective metabolites such as KYNA. We further hypothesized that these responses would be moderated by exercise‐related factors and baseline population characteristics, particularly the presence of clinical conditions associated with chronic inflammation or altered KYN pathway metabolism.

## 2 Methods

### 2.1 Eligibility Criteria and Research Sources

This systematic review was conducted following the Preferred Reporting Items for Systematic Reviews and Meta‐Analysis Protocols (PRISMA) guidelines [37] and was prospectively registered in the International Prospective Register of Systematic Reviews (PROSPERO) database under number CRD42022351481.

The search was conducted from inception until June 2, 2026, in MEDLINE (via PubMed), Web of Science, and Scopus databases. No date restrictions were applied, and filters were set for human studies and English language. A Boolean search strategy using terms related to “physical exercise” and “kynurenine pathway” was applied to identify relevant trials (Data S1).

Only original trials investigating the effects of a single exercise session on KYN pathway metabolites were included. Studies were considered if they met the following PICOS criteria (Population, Intervention, Comparison, Outcome and Study design): (P) adults (≥ 18 years); (I) clinical trials involving a single exercise session; (C) before and after exercise; (O) evaluation of at least two biomarkers of the KYN pathway, including TRP and its downstream metabolites; and (S) controlled or uncontrolled trials.

Trials that included dietary or supplementation interventions affecting the KYN pathway were excluded. Longitudinal studies, study protocols, case reports, epidemiological studies, reviews, and editorials were also not included. For quantitative synthesis, studies were required to provide sufficient data (mean and standard deviation or equivalent dispersion measures) to calculate effect sizes for pre–post changes. Only plasma or serum assessments obtained within 3 h after exercise cessation were considered for quantitative synthesis.

### 2.2 Study Selection

Two investigators (M.V.S.R. and J.P.B.) independently screened potential studies in two stages: (1) title and abstract review, and (2) full‐text evaluation. Studies that did not meet the inclusion criteria at any stage were excluded. Records were imported into an electronic reference manager, and duplicate entries were removed prior to screening. Reference lists of the included studies were manually screened to identify additional eligible studies. Discrepancies were resolved through discussion between investigators, and if consensus could not be reached, a third reviewer (G.A.P.) was consulted. Agreement on inclusion was validated in a random sample of 50 abstracts, yielding a Cohen's kappa coefficient of 0.84–0.99 (*p* < 0.05).

### 2.3 Methodological Quality and Risk of Bias Assessment

Methodological quality was independently assessed by two reviewers (MR and JB), with disagreements resolved by consensus, using the Tool for the Assessment of Study Quality and Reporting in Exercise (TESTEX) scale [38]. TESTEX is a 15‐point instrument developed for exercise studies, including 5 points for study quality and 10 for reporting. Because the scale was originally designed for longitudinal training interventions, three criteria were considered not applicable to the present review of acute exercise studies: criterion 7 (intention‐to‐treat analysis), criterion 10 (monitoring of the control group over time), and criterion 11 (maintenance of constant exercise intensity). Therefore, the maximum possible score was reduced from 15 to 12.

Risk of bias was assessed by the same reviewers using the Cochrane Risk of Bias in Non‐randomized Studies of Interventions tool (ROBINS‐I), which evaluates internal validity across seven domains: confounding, participant selection, intervention classification, deviations from intended interventions, missing data, outcome measurement, and selective reporting [39].

### 2.4 Data Extraction and Quantitative Synthesis

Data were independently extracted by two reviewers (M.V.S.R. and J.P.B.) using a standardized form. Extracted data included participant characteristics, exercise protocol variables (modality, intensity, and duration), sampling time points, and quantitative data for KYN pathway metabolites (TRP, KYN, QA, KYNA, and its ratios). Mean and standard deviation values before and after exercise were obtained for each outcome, and corresponding authors were contacted when data were missing.

Because analytical platforms and reporting formats varied across studies, metabolite concentrations were extracted as reported by the original studies and quantitative synthesis was performed using standardized pre–post effect sizes (Hedges' g). Gene‐expression and protein‐level findings were extracted qualitatively and were not quantitatively combined with metabolite outcomes into a composite score.

Each exercise condition was treated as an independent intervention. When studies included multiple participant subgroups, effect sizes were calculated separately for each subgroup.

### 2.5 Data Synthesis and Outcome Handling

Separate meta‐analyses were conducted for KYN pathway metabolites and, when available, metabolite‐derived ratios. Outcomes were categorized into two predefined temporal windows: immediate post‐exercise responses (≤ 5 min after exercise cessation) and recovery responses (> 5 min up to 3 h after exercise). Separate meta‐analyses were performed for each temporal window whenever sufficient data were available. When multiple post‐exercise time points were reported, effect sizes were calculated for each time point and subsequently combined into a single estimate per study using standard meta‐analytic procedures for dependent effect sizes, thereby avoiding overweighting studies with repeated measurements.

### 2.6 Statistical Analysis

Statistical significance was set at *p* ≤ 0.05. Meta‐analyses were performed using Comprehensive Meta‐Analysis Software (version 4, Biostat Inc., Englewood, NJ, USA) when at least three independent studies were available for a given metabolite or ratio. Effect sizes were calculated as standardized mean differences (Hedges' g) for pre–post changes in KYN pathway metabolites, with variance estimates adjusted for within‐subject correlation. When this correlation was not reported, a conservative value of *r* = 0.5 was assumed.

Random‐effects models (DerSimonian–Laird) with inverse‐variance weighting were used because of the expected methodological and clinical heterogeneity across studies. Heterogeneity was assessed using the *I* ^2^ statistic, with values of 25%, 50%, and 75% indicating low, moderate, and high heterogeneity, respectively [40, 41].

Prespecified subgroup analyses were conducted according to population characteristics (healthy vs. clinical), physical fitness (trained vs. untrained), and exercise modality (aerobic vs. resistance), based on classifications reported by the original study authors. Differences between “gs” obtained for subgroups were tested by the Q‐test based on the analog to the analysis of variance. Sensitivity analyses excluding studies with serious/critical ROBINS‐I ratings or outlying effect sizes were performed to test the robustness of pooled estimates. Publication bias was assessed by visual inspection of funnel plots, Egger's regression test, and trim‐and‐fill procedures when appropriate.

## 3 Results

Figure 1 presents the PRISMA flow diagram for study selection. Of the 3203 records identified, 1038 duplicates were removed and 2115 were excluded after title and abstract screening, leaving 50 articles for full‐text assessment. Of these, 26 were excluded for explicit reasons: 17 evaluated exercise‐training rather than a single exercise session, six assessed fewer than two KYN pathway biomarkers, two involved supplementation affecting the KYN pathway, and one lacked pre‐ and post‐exercise results. Thus, 24 met the inclusion criteria for the systematic review, and 20 provided sufficient quantitative data for meta‐analysis.

**FIGURE 1:**
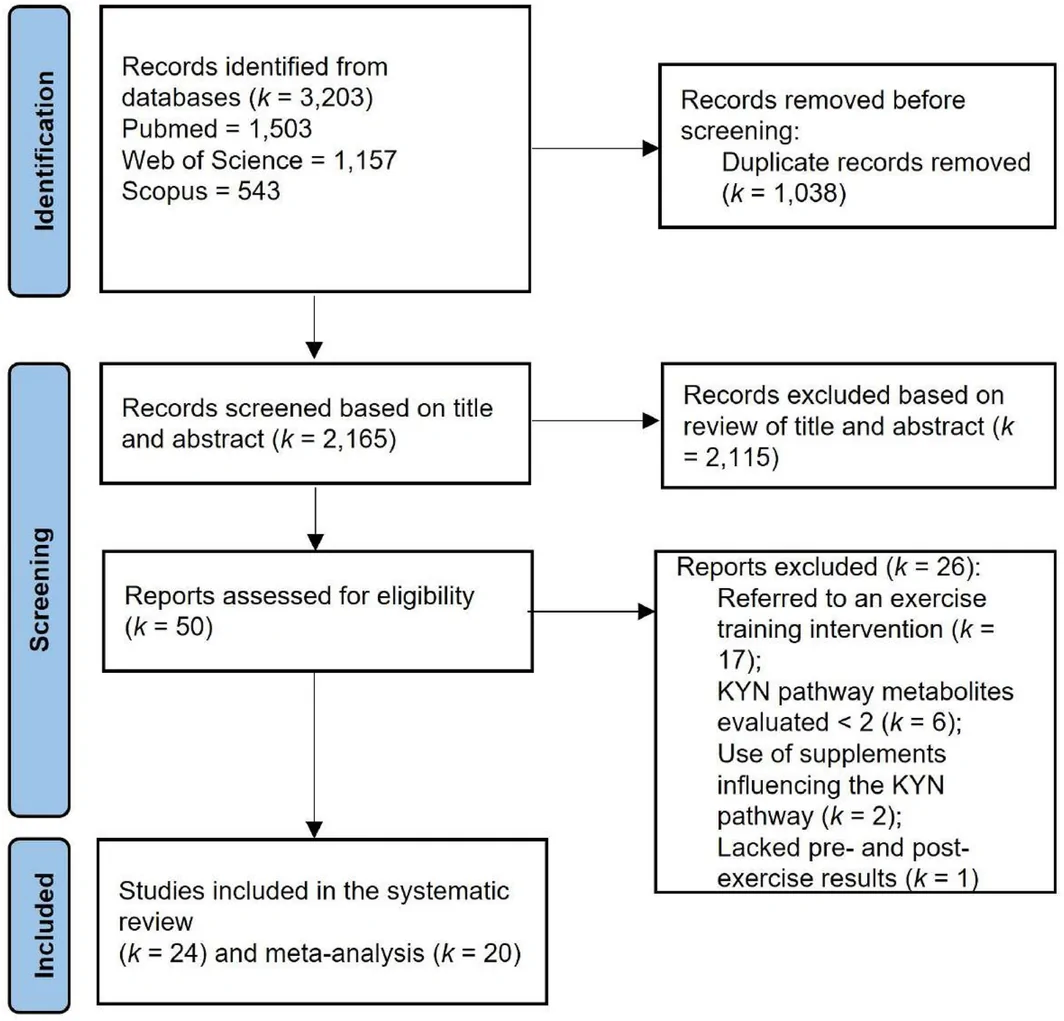
PRISMA flow diagram summarizing the search and selection of articles.

### 3.1 Study Quality and Risk of Bias

Table 1 presents the methodological quality scores based on the TESTEX scale. Overall, methodological quality was moderate, with TESTEX scores between 4 and 11 (median score 7). Eight studies scored ≥ 9 points [19, 24, 25, 31, 44, 48, 50, 51], 14 scored 5–8 points [26, 27, 28, 31, 32, 33, 34, 43, 45, 46, 47, 49, 52, 53], and two scored ≤ 4 points [29, 30].

**TABLE 1:** TESTEX assessment of the quality and reporting of studies investigating acute exercise‐induced modulation of the kynurenine pathway in health and disease.

Study	Study quality criterion					Study reporting criterion							∑ (MAX 12)^a^
	1	2	3	4	5	6	7	8	9	10	11	12	
Lewis 2010 [28]	1	0	0	1	0	2	NA	0	1	NA	NA	0	5
Strasser 2016a [31]	1	0	0	0	0	2	NA	0	1	NA	NA	1	5
Strasser 2016b [42]	1	1	1	1	1	2	NA	2	1	NA	NA	1	11
Schlittler 2016 [30]	1	0	0	0	0	2	NA	0	1	NA	NA	0	4
Baxter‐Parker 2019 [25]	1	1	0	1	0	2	NA	2	1	NA	NA	1	9
Herhaus 2021 [32]	1	1	0	1	0	2	NA	0	1	NA	NA	1	7
Isung 2021 [43]	1	0	0	0	0	2	NA	0	1	NA	NA	1	5
Joisten 2020a [19]	1	1	0	1	0	2	NA	2	1	NA	NA	1	9
Joisten 2020b [24]	1	1	0	1	0	2	NA	2	1	NA	NA	1	9
Joisten 2024 [44]	1	1	0	1	0	2	NA	2	1	NA	NA	1	9
Jonsson 2022 [33]	0	1	0	1	0	2	NA	0	1	NA	NA	1	6
Trepci 2020 [45]	0	0	0	0	0	2	NA	2	1	NA	NA	1	6
Umlauff 2021 [46]	1	0	0	1	0	2	NA	2	1	NA	NA	1	8
Koliamitra 2019 [26]	1	0	0	0	0	2	NA	0	1	NA	NA	1	5
Mieszkowski 2022 [29]	1	0	0	0	0	2	NA	0	1	NA	NA	0	4
Louvrou 2025 [47]	1	1	0	1	1	2	NA	0	1	NA	NA	1	8
Kuang 2025 [27]	1	0	0	0	0	2	NA	0	1	NA	NA	1	5
Mudry 2016 [34]	1	0	0	0	0	2	NA	2	1	NA	NA	1	7
Bansi 2018 [48]	1	1	0	1	1	2	NA	2	1	NA	NA	0	9
Donia 2019 [49]	1	0	0	0	0	2	NA	0	1	NA	NA	1	5
Schenk 2020 [50]	1	1	0	1	0	2	NA	2	1	NA	NA	1	9
Joisten 2021 [51]	1	1	0	1	1	2	NA	2	1	NA	NA	1	10
Javelle 2025 [52]	1	0	0	1	0	2	NA	2	1	NA	NA	1	8
Swaminathan 2026 [53]	1	0	0	0	0	2	NA	0	1	NA	NA	1	5

Abbreviation: ∑ = Sum of all criteria.

^a^ Total out of 12 points; items 7, 10, and 11 not applied (NA).

Risk of bias assessed with the ROBINS‐I tool is shown in Data S2. Most studies (*k* = 21) presented low risk of bias in domains related to deviations from intended interventions, missing data, outcome measurement, and selective reporting. In contrast, concerns were more frequent for confounding, participant selection, and intervention classification, with several studies (*k* = 18) rated as having moderate or serious risk of bias. Overall risk of bias ranged from low to critical, indicating that the main methodological limitations were related to study design and baseline comparability rather than outcome assessment or reporting.

### 3.2 Study Characteristics and Major Outcomes

Table 2 summarizes the main characteristics of the studies included in the review, including participant characteristics, exercise interventions, and primary outcomes. Of the 24 included studies, 17 studies involved healthy participants [19, 24, 25, 26, 27, 28, 29, 30, 31, 32, 33, 42, 43, 44, 45, 46, 47], and 7 studies included populations with chronic conditions, including multiple sclerosis [48, 49, 51], incomplete spinal cord injury [49] type 2 diabetes [34], heart failure with reduced ejection fraction [53], prostate cancer [50], and borderline personality disorder [52].

**TABLE 2:** Summary of studies included in the systematic review.

Study	Sample	Exercise intervention	Time points	Main results		Conclusion
				Time effect	Condition effect	
Lewis et al. (2010) [28]	Healthy adults performing a treadmill exercise (*n* = 70) or a cycle ergometer test (*n* = 8); Healthy amateur marathon runners (*n* = 25, 19 males and 6 females)	Exercise treadmill test (ETT) or cycle ergometer test (CPET); Marathon race	ETT and CPET: before, peak exercise, and 60 min post‐exercise Marathon: before and immediately post‐race	ETT and CPET: KYN pathway metabolites ↔ Marathon: KYN ↑ TRP ↓ QA ↑ AA ↑	NA	Prolonged strenuous exercise activates the KYN pathway, increasing neuroactive metabolites and reducing TRP, likely driven by metabolic and inflammatory stress. Short acute exercise induces a less pronounced effect on KYN metabolism compared to prolonged exertion
Strasser et al. (2016a) [31]	Trained athletes (*n* = 33, 16 males and 17 females)	Incremental exercise test on a cycle ergometer, followed by a 20‐min rest period, and then a 20‐min maximal time‐trial on the cycle ergometer	Before and 5 min post‐exercise	TRP ↓ KYN ↑ KYN/TRP ↑	NA	Exhaustive exercise significantly reduced TRP concentrations by 12% and increased KYN levels by 6%
Strasser et al. (2016b) [42]	Trained athletes: Placebo group (*n* = 15)	Incremental exercise test on a cycle ergometer, followed by a 20‐min rest period, and then a 20‐min maximal time‐trial on the cycle ergometer	Before and 5 min post‐exercise at baseline and after 12 weeks of training	Placebo (baseline PRE vs. POST) TRP ↔ KYN ↔ KYN/TRP ↑ Placebo (12 weeks PRE vs. POST) TRP ↓ KYN ↔ KYN/TRP ↑	NA	Acute exhaustive exercise decreased serum TRP levels and increased the KYN/TRP ratio in the placebo group
Schlittler et al. (2016) [30]	Recreationally active subjects: 150‐km road cycling time trial (*n* = 9, all male); Half‐marathon race (*n* = 11; 10 males, 1 female); Eccentric muscle work (*n* = 9, all male)	150‐km road cycling time trial Half‐marathon race on a hilly course Eccentric exercise that consisted of a series of 100 drop jumps from a 50‐cm platform with active deceleration to 90° knee angle, immediately followed by a maximal rebound jump	Before and 1, 5, 24 h, and 7 days post‐150‐km cycling; Before and 30 min post‐half‐marathon; Before and 1, 24, and 48 h post‐eccentric exercise	150‐km cycling: At 1 h: QA ↑ KYNA ↑ QA/KYNA ↓ All other time‐points: QA ↔ KYNA ↔ QA/KYNA ↔ Half‐marathon race KYNA ↑ 100 drop jumps: All time‐points: QA ↔ KYNA ↔ QA/KYNA ↔	NA	Plasma concentration of KYNA increases and the QA/KYNA ratio decreases transiently following prolonged endurance exercise but not after eccentric muscle work
Baxter‐Parker et al. (2019) [25]	Healthy adults (*n* = 43; 23 males and 20 females)	Incremental exercise test on a cycle ergometer (exercise session) or 10 min resting (control session)	Before and 30, 60, and 120 min post‐ exercise or resting	Exercise session: TRP ↓ KYN ↔ KYN/TRP ↑ Control session: TRP ↓ KYN↔ KYN/TRP ↑	Exercise vs. Control: TRP ↔ KYN ↔ KYN/TRP ↔	Both sessions reduced plasma TRP levels over the measurement period, which may be due to minor IDO activation or natural circadian rhythmic variations seen throughout the day
Herhaus et al. (2021) [32]	Healthy men (*n* = 35)	Multiple Wingate all‐out tests: 10‐min warm‐up at 70 rpm + 4 × 30 s at 120 rpm in isokinetic mode, with 7.5 min active recovery at 70 rpm	Before (−1 min), immediately after (+1 min), and 30 and 60 min post‐exercise	TRP ↓ KYN ↑ KYN/TRP ↑ QA ↑ KYNA ↑ QA/KYN ↑ KYNA/KYN ↑ QA/KYNA ↓	NA	An exercise‐induced increase in metabolic flux toward KYNA was observed in response to both physical and psychological stressors, but the effect was significantly greater following physical stress
Isung et al. (2021) [43]	Healthy trained adults (*n* = 14, 6 males and 8 females)	Four consecutive days of running: Days 1 and 3: 30‐min high‐intensity interval training (10 × 1‐min high intensity, RPE 18–19, with 2‐min low intensity, RPE 9–11); Days 2 and 4: ≥ 60‐min continuous running at vigorous intensity, RPE 14–16	Plasma and cerebrospinal fluid collected before and 24 h post‐exercise sessions	In plasma: TRP ↓ KYN ↓ KYNA ↔ 3HK ↔ PIC ↔ QA ↔ KYN/TRP ↔ PIC/QA ↔ In cerebrospinal fluid: TRP ↔ KYN ↔ KYNA ↑ 3HK ↑ PIC ↑ QA ↔ KYN/TRP ↔ PIC/QA ↔	NA	Acute aerobic physical exercise affects KYN pathway metabolites systemically and in the cerebrospinal fluid
Joisten et al. (2020a) [19]	Healthy men (*n* = 12)	Strength exercise: 5 sets with 10 repetitions at 80% of 1RM (HYP) or 15 sets with 1RM (MAX)	Before, immediately after, and 1 h post‐exercise	HYP: TRP ↔ KYN ↔ KYN/TRP ↔ QA ↔ QA/KYN ↔ KYNA ↔ KYNA/KYN ↑ (immediately after exercise) QA/KYNA ↔ MAX: TRP ↔ KYN ↔ KYN/TRP ↔ QA ↔ QA/KYN ↔ KYNA ↔ KYNA/KYN ↔ QA/KYNA ↔	HYP vs. MAX: TRP ↔ KYN ↔ KYN/TRP ↔ QA ↔ QA/KYN ↔ KYNA ↑ (immediately after exercise) KYNA/KYN ↔ QA/KYNA ↓ (immediately after exercise)	Although there was no change in the initial activation of the KYN pathway following either condition, the downstream kinetics differed transiently between HYP and MAX
Joisten et al. (2020b) [24]	Healthy men (*n* = 24)	Endurance exercise (EE): 5‐min warm‐up +45‐min cycling at 60% peak power output Resistance exercise (RE): 5‐min warm‐up +45 min, 5 exercises each 4 × 8–10 repetitions at 70% of 1 RM	Before, immediately after, and 1 h post‐exercise	EE: TRP ↔ KYN ↔ KYN/TRP ↔ KYNA ↑ (immediately after exercise) KYNA/KYN ↑ QA ↑ (immediately after exercise) QA/KYN ↔ QA/KYNA ↔ IDO1 gene expression in PBMCs ↔ KAT4 gene expression in PBMCs ↑ (immediately after exercise) RE: TRP ↓ (immediately after exercise) KYN ↔ KYN/TRP ↑ (immediately after exercise) KYNA ↔ KYNA/KYN ↔ QA ↔ QA/KYN ↔ QA/KYNA ↔ IDO1 gene expression in PBMCs ↔ KAT4 gene expression in PBMCs ↔	EE vs. RE: TRP ↔ KYN ↔ KYN/TRP ↔ KYNA ↑ KYNA/KYN ↑ QA ↔ QA/KYN ↑ QA/KYNA ↔ IDO1 gene expression in PBMCs ↔ KAT4 gene expression in PBMCs ↔	There was a stronger activation of the KYN pathway induced by EE compared to RE, particularly a greater conversion of KYN to KYNA in response to EE
Joisten et al. (2024) [44]	Healthy recreational runners (*n* = 24, 12 males and 12 females)	High‐intensity interval exercise (HIIE): 7‐min warm‐up at 70% VO_2max_ + 6 × 3‐min of running at 90% VO_2max_ with 3‐min active recovery at 90% VO_2max_ + 7‐min cool‐down at 70% VO_2max_ Moderate‐intensity continuous exercise (MICE): 50‐min running at 70% VO_2max_	Before, immediately after, and 1 h post‐exercise	HIIE: TRP ↔ KYN ↑ (immediately after exercise) KYNA ↑ QA ↑ IDO1 gene expression in PBMCs ↑ IDO2 gene expression in PBMCs ↔ KAT3 gene expression in PBMCs ↑ (1 h after exercise) KAT4 gene expression in PBMCs ↔ Gene expression levels of AhR target genes in PBMCs ↑ Protein levels of AhR targets in PBMCs ↔ MICE: TRP ↔ KYN ↑ (immediately after exercise) KYNA ↑ QA ↑ IDO1 gene expression in PBMCs ↑ (1 h after exercise) IDO2 gene expression in PBMCs ↑ (immediately after exercise) KAT3 gene expression in PBMCs ↑ (1 h after exercise) KAT4 gene expression in PBMCs ↑ (immediately after exercise) Gene expression levels of AhR target genes in PBMCs ↑ Protein levels of AhR targets in PBMCs ↔	HIIE vs. MICE: TRP ↔ KYN ↔ KYNA ↑ QA ↑ IDO1 gene expression in PBMCs ↔ IDO2 gene expression in PBMCs ↔ KAT3 gene expression in PBMCs ↔ KAT4 gene expression in PBMCs ↓ (immediately after exercise) Gene expression levels of AhR target genes in PBMCs ↑ Protein levels of key AhR targets in PBMCs ↔	The KYN–AhR axis in circulating immune cells responds to acute exercise, with higher‐intensity training producing stronger immunomodulatory effects, even when exercise duration and energy expenditure are matched
Jonsson et al. (2022) [33]	Healthy adults: Placebo group (*n* = 10, 7 males and 3 females)	10‐min warm‐up +60‐min dynamic one‐leg knee extension exercise at 70% W_max_ at 50–60 rpm	Blood samples: before, during, immediately after, and up to 180 min post‐exercise Muscle biopsies: before, immediately after, and 90‐ and 180‐min post‐exercise	In plasma: TRP ↓ (from 90‐min up to 180‐min post‐exercise) KYN ↓ (from 30‐min up to 180‐min post‐exercise) KYNA ↔ In muscle: TRP ↔ KYN ↓ (180‐min post‐exercise) KAT4 mRNA or total protein expression ↔	NA	The plasma levels of TRP and KYN decreased, while KYNA remained essentially unchanged; these effects were not attributable to alterations in KYN metabolism in skeletal muscle
Trepci et al. (2020) [45]	Healthy recreationally active men: Young (*n* = 10) and old (*n* = 10)	8‐min warm‐up at W equal to the individual's body mass + 6 × 30‐s of all‐out cycling at 0.7 Nm/kg body weight with 4‐min passive recovery between sprints	Before, 1‐h and 24‐h post‐exercise	Young group: TRP ↔ KYN ↔ KYNA ↔ 3HK ↔ QA ↔ PIC ↔ Old group: TRP ↔ KYN ↑ (1‐h post‐exercise) KYNA ↑ (24‐h post‐exercise) 3HK ↔ QA ↔ PIC ↔	Young vs. Old (only compared at rest): TRP ↔ KYN ↔ KYNA ↔ 3HK ↔ QA ↔ PIC ↔	There is an age‐dependent effect on the degree to which a single intense exercise session affects KYN metabolite levels
Umlauff et al. (2021) [46]	Healthy trained young women: Oral contraceptive users (OC; *n* = 8) and naturally menstrual cycling women (MC; *n* = 13)	4 × 10 repetitions back squat at 70% maximal strength with 2‐min interset rest	Before, immediately after, and 24‐h post‐exercise	OC group: TRP ↓ (24‐h post‐exercise) KYN ↔ KYN/TRP ↔ MC group: TRP ↓ (24‐h post‐exercise) KYN ↔ KYN/TRP ↔	OC vs. MC: TRP ↔ KYN ↔ KYN/TRP ↔	TRP decreased 24 h after acute strength exercise, whereas KYN and KYN/TRP remained unchanged
Koliamitra et al. (2019) [26]	Healthy older women (*n* = 19)	Incremental exercise test on a cycle ergometer	Before and 1‐min post‐exercise	TRP ↔ KYN ↑ KYN/TRP ↑	NA	Endurance exercise activates the kynurenine pathway in older women
Mieszkowski et al. (2022) [29]	Healthy amateur male runners: Placebo group (*n* = 19)	Ultramarathon	Before, immediately after, and 24‐h post‐run.	TRP ↔ KYN ↑ 3HK ↑ (immediately after exercise) KYNA ↑ (immediately after exercise) XA ↑ (immediately after exercise) QA ↑ (immediately after exercise) PIC ↑ (immediately after exercise) KYN/TRP ↑ (immediately after exercise) KYNA/KYN ↑ (immediately after exercise) KYNA/QA ↔ PIC/QA ↔	NA	Ultramarathon increased all measured KYN metabolites
Louvrou et al. (2025) [47]	Young healthy males (*n* = 11)	Cycling (10‐min warm‐up at 50 W followed by exertional heating via exercise at 60% of VO_2max_, until rectal temperature reached 39°C)	Before, immediately after, and until rectal temperature returned to 37.5°C	TRP ↔ KYN ↓ QA ↑ KYNA ↑ 3HK ↑ PIC ↑ KYN/TRP ↑ QA/KYNA ↓	NA	Acute exercise increased the KYN/TRP ratio and KYNA, QA, PIC, and 3HK metabolites
Kuang et al. (2025) [27]	Healthy males (*n* = 10)	Aerobic exercise: running (warm‐up of 3 laps of a 400‐m track followed by 45 min at 60%–70% HR_max_) Resistance exercise: 5‐kg barbell, 3 sets of 15 repetitions of each exercise with 60 s rest between sets, 60%–70% HR_max_ for 45 min	Before, 30 min, and 24‐h post‐exercise	Aerobic: NR Resistance: 30 min after: AA ↓ Formylkynurenine ↑ 24 h after: AA ↑ Formylkynurenine NR	NA	Resistance exercise increased formylkynurenine and activated TRP metabolism, suggesting involvement of the KYN pathway during recovery
Mudry et al. (2016) [34]	Men with type 2 diabetes (T2D; *n* = 12); Men with normal glucose tolerance (NGT; *n* = 12)	30‐min cycling at 85% HR_max_	Before, immediately after, and 3 h post‐exercise	T2D: TRP ↓ KYN ↓ KYNA ↑ KYNA/KYN ↑ KAT4 mRNA muscle expression ↓ PGC‐1α1 mRNA muscle expression ↑ KAT1, KAT2, KAT3, PPARα and PPARδ mRNA muscle expression ↔ NGT: TRP ↓ KYN ↓ KYNA ↑ KYNA/KYN ↑ KAT4 mRNA muscle expression ↓ PGC‐1α1 mRNA muscle expression ↑ KAT1, KAT2, KAT3, PPARα and PPARδ mRNA muscle expression ↔	T2D vs. NGT: Serum KYN pathway metabolites ↔ KAT1, KAT2, and PPARα mRNA muscle expression ↓ KAT3, KAT4, PPARδ and PGC‐1α1 mRNA muscle expression ↔	Acute exercise reduced circulating levels of TRP and KYN, while increasing KYNA. Acute exercise‐induced changes in KYN metabolism were similar in patients with type 2 diabetes
Bansi et al. (2018) [48]	Patients with multiple sclerosis (MS): Secondary progressive MS (SPMS; *n* = 24); Relapsing–remitting MS (RRMS; *n* = 33)	Incremental exercise test on a cycle ergometer	Before and immediately post‐exercise	SPMS: 5HT ↔ TRP ↑ KYN ↑ KYN/TRP ↓ RRMS: 5HT ↔ TRP ↓ KYN ↑ KYN/TRP ↑	SPMS vs. RRMS: 5HT ↔ TRP ↑ KYN ↔ KYN/TRP ↓	MS phenotypes have different KYN responses to acute maximal exercise
Donia et al. (2019) [49]	Patients with multiple sclerosis or spinal cord injury (*n* = 13, 3 males and 10 females)	30‐min exercise at 60% of VO_2max_ on a recumbent cross‐trainer	Before, immediately after, and 1 h post‐exercise	TRP ↔ KYN ↔ KYN/TRP ↔	NA	Acute aerobic exercise did not significantly change TRP, KYN, or KYN/TRP
Schenk et al. (2020) [50]	Prostate cancer survivors: Intervention group (*n* = 11) and control group (*n* = 13)	5‐min warm‐up +20‐min of cycling at 75% VO_2peak_ + 5‐min cool‐down (intervention group) or 30 min resting (control group)	Before and immediately post‐exercise	Intervention group: TRP ↔ KYN ↔ QA ↔ KYNA ↔ KYN/TRP ↔ QA/KYN ↔ KYNA/KYN ↔ QA/KYNA ↔ Control group: TRP ↑ KYN ↔ QA ↔ KYNA ↔ KYN/TRP ↓ QA/KYN ↔ KYNA/KYN ↔ QA/KYNA ↔	Intervention vs. Control: TRP ↓ KYN ↔ QA ↔ KYNA ↔ KYN/TRP ↑ QA/KYN ↔ KYNA/KYN ↔ QA/KYNA ↔	A single bout of aerobic exercise reduced TRP levels and increased the KYN/TRP ratio
Joisten et al. (2021) [51]	Patients with multiple sclerosis: Moderate continuous training (MCT; *n* = 34); High‐intensity interval training (HIIT; *n* = 35)	MCT: 3‐min warm‐up +24‐min of cycling at 65% HR_max_ + 3‐min cool‐down HIIT: 3‐min warm‐up +5 × 1.5‐min of cycling at 95%–100% HR_max_ with 2‐min active recovery +3‐min cool‐down	Before, immediately after, and 3 h post‐exercise	MCT: TRP ↓ (3 h after exercise) KYN ↓ (3 h after exercise) KYN/TRP ↔ QA ↔ QA/KYN ↔ KYNA ↓ (3 h after exercise) KYNA/KYN ↔ QA/KYNA ↑ (3 h after exercise) HIIT: TRP ↓ KYN ↓ (t_2_) KYN/TRP ↔ QA ↔ QA/KYN ↔ KYNA ↑ (immediately after exercise) KYNA/KYN ↑ (immediately after exercise) QA/KYNA ↓ (immediately after exercise)	MCT vs. HIIT: TRP ↔ KYN ↔ KYN/TRP ↔ QA ↔ QA/KYN ↔ KYNA ↓ KYNA/KYN ↔ QA/KYNA ↔	Greater increases in KYNA levels after acute exercise were observed following HIIT than MCT at both post‐exercise time points
Javelle et al. (2025) [52]	Patients with borderline personality disorder (BPD; *n* = 14); healthy control group (*n* = 9).	5‐min warm‐up +20‐min of cycling at 64%–76% HR_max_ or > 77% HR_max_ + 4‐min cool‐down or 30 min‐resting (control session)	Before and after each condition (within 5–15 min)	BPD group: TRP ↔ KYN ↔ QA ↔ KYNA ↑ KYNA/KYN ↑ KYNA/QA ↑ IL‐6 ↑ Control group: NR	BPD versus Control: NR	BPD patients presented a peripheral metabolic shift toward the neurotoxic branch of the KYN pathway compared to healthy controls. This imbalance may be mitigated through increased activity in the neuroprotective KYN branch induced by physical exercise
Swaminathan et al. (2026) [53]	Patients with heart failure with reduced ejection fraction (HFrEF, *n* = 16) and healthy controls (*n* = 13)	Incremental exercise test on a cycle ergometer	Before, immediately after, and 2 h post‐exercise	Patients with HFrEF group: TRP ↓ KYN/TRP ↑ KYNA/TRP ↑ QA/TRP ↑ Control group: TRP ↓ KYN/TRP ↑ KYNA/TRP ↑ QA/TRP ↑	NA	A single acute exercise bout reduced plasma TRP levels and increased KYN pathway activity, reflected by elevated KYN/TRP and KYNA/TRP ratios; HFrEF patients additionally exhibited delayed accumulation of QUIN during recovery

Abbreviations: 3HK, 3‐hydroxykynurenine; 5HT, 5‐hydroxytryptamine; AA, anthranilic acid; AhR, aryl hydrocarbon receptor; HR_max_, maximal heart rate; IDO, indoleamine 2,3‐dioxygenase; IL‐6, interleukin‐6; KAT, kynurenine aminotransferase; KYN, kynurenine; KYNA, kynurenic acid; mRNA, messenger ribonucleic acid; NA, not applicable; NR, not reported; PBMC, peripheral blood mononuclear cells; PGC‐1α1, peroxisome proliferator‐activated receptor gamma coactivator 1‐alpha 1; PIC, picolinic acid; PPARα, peroxisome proliferator‐activated receptor alpha; PPARδ, peroxisome proliferator‐activated receptor delta; QA, quinolinic acid; RM, repetition maximum; RPE, rate of perceived exertion; rpm, rounds per minute; TDO, tryptophan‐2,3‐dioxygenase; TRP, tryptophan; VO_2peak_, peak oxygen consumption; W_max_, maximal wattage; XA, xanthurenic acid.

Most trials investigated aerobic exercise (*k* = 23), whereas only four studies additionally included or exclusively assessed resistance exercise interventions [19, 24, 27, 46]. Acute exercise‐induced changes in KYN pathway metabolites were reported in 22 studies, while two studies found no significant metabolite alterations following exercise [19, 46].

Regarding pathway‐specific responses, 8 studies reported metabolite patterns consistent with increased peripheral conversion of KYN toward KYNA [24, 30, 32, 33, 34, 43, 45, 48], whereas 12 studies observed increases in KYN and QA [24, 25, 26, 27, 28, 29, 30, 31, 32, 42, 48, 53]. Additionally, 4 studies reported activation of both pathway branches for the same stimulus [29, 32, 44, 47]. Furthermore, some intermediate metabolites, such as picolinic acid (PIC) and 3‐hydroxykynurenine (3HK), have been less extensively investigated, with data available from only four studies [29, 43, 45, 47]. However, quantitative synthesis was not performed for these metabolites because only two studies provided extractable pre–post exercise data within the prespecified time window (≤ 3 h post‐exercise) [29, 47]. Evidence for other metabolites was even more limited, with only one study reporting changes in xanthurenic acid [29] and anthranilic acid [28], respectively.

### 3.3 Effect Size Estimates

A total of 20 studies (9–25 interventions, depending on the outcome) were included in the meta‐analysis. Figures 2, 3, 4 present the forest plots for the immediate post‐exercise responses (≤ 5 min after exercise cessation) for TRP and KYN (Figure 2), QA and KYNA (Figure 3), and the KYN/TRP and QA/KYNA ratios (Figure 4), respectively. Corresponding forest plots for the recovery phase (> 5 min up to 3 h after exercise) are available in Data S3.

**FIGURE 2:**
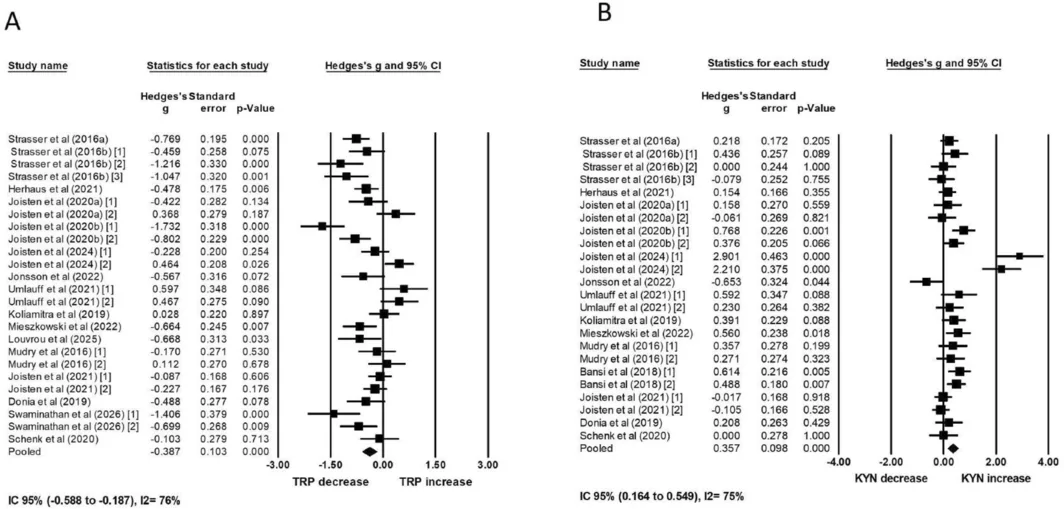
Hedges' g pooled effect sizes and 95% confidence intervals for the immediate post‐exercise responses (≤ 5 min after exercise cessation) on TRP (Panel A, *k* = 16, 25 interventions) and KYN (Panel B, *k* = 15, 24 interventions). Numerals between brackets represent different interventions within the same study.

**FIGURE 3:**
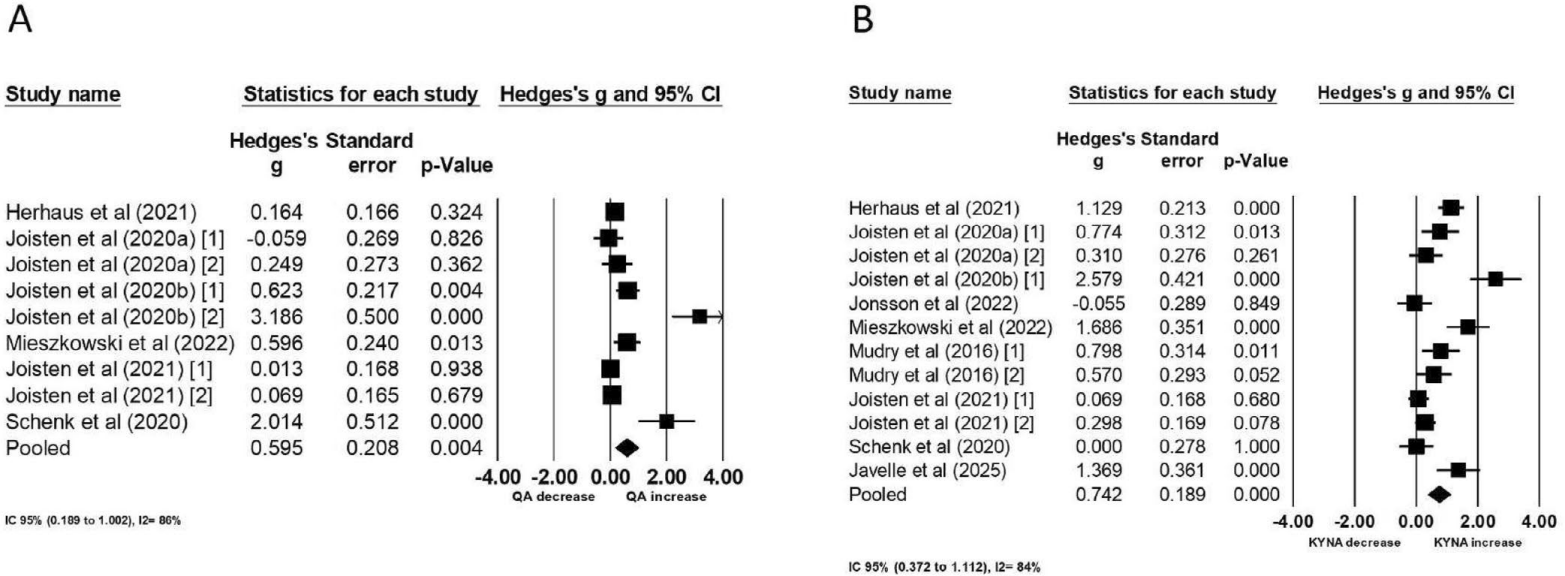
Hedges' g pooled effect sizes and 95% confidence intervals for the immediate post‐exercise responses (≤ 5 min after exercise cessation) on QA (Panel A, *k* = 6, 9 interventions) and KYNA (Panel B, *k* = 9, 12 interventions). Numerals between brackets represent different interventions within the same study.

**FIGURE 4:**
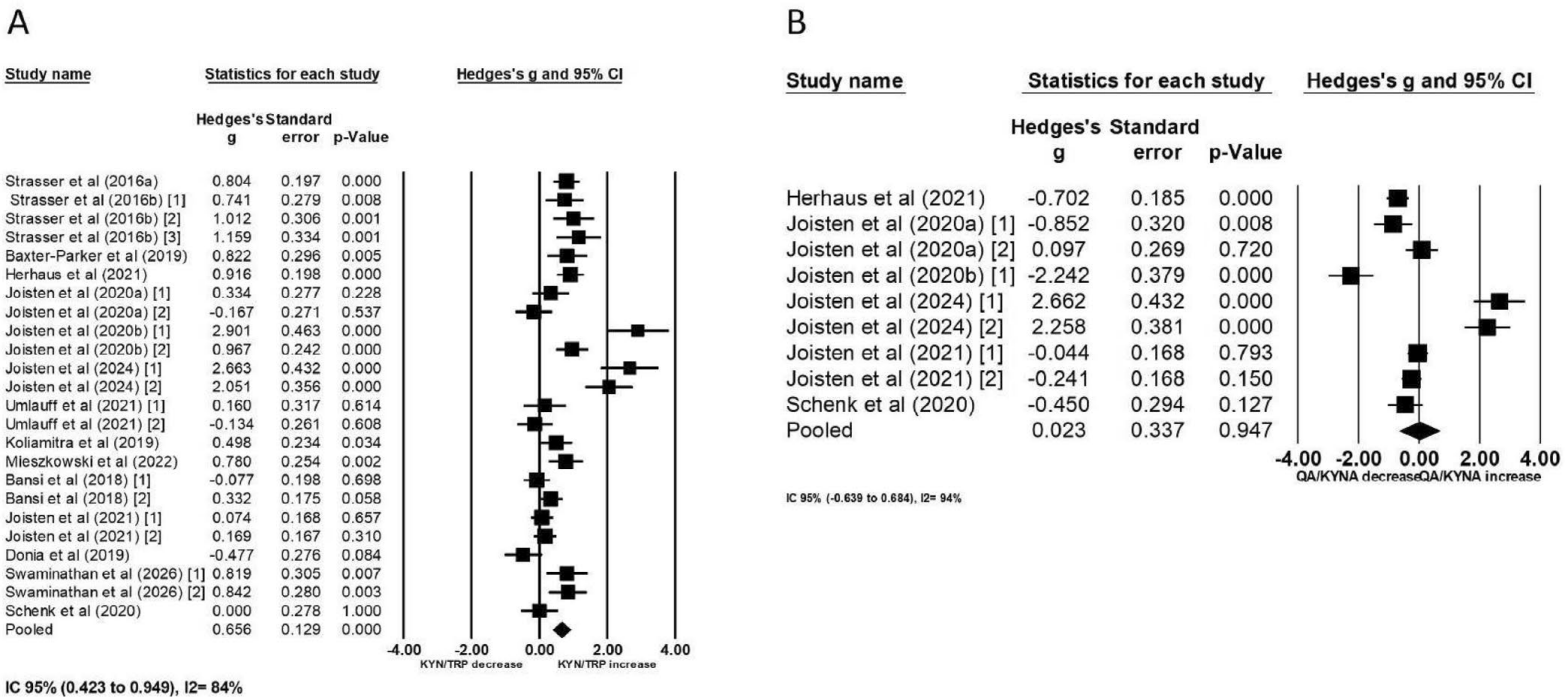
Hedges' g combined effect sizes and 95% confidence intervals for the immediate post‐exercise responses (≤ 5 min after exercise cessation) on KYN/TRP (Panel A, *k* = 15, 24 interventions) and QA/KYNA (Panel B, *k* = 6, 9 interventions). Numerals between brackets represent different interventions within the same study.

For the immediate post‐exercise responses, exercise significantly reduced circulating TRP concentrations (*g* = −0.387; 95% CI −0.588 to −0.187; *p* < 0.001; *I* ^2^ = 76%) (Figure 2A) and increased KYN (*g* = 0.357; 95% CI 0.164–0.549; *p* < 0.001; *I* ^2^ = 75%) (Figure 2B). Moreover, QA (*g* = 0.595; 95% CI 0.189–1.002; *p* = 0.004; *I* ^2^ = 86%) (Figure 3A), KYNA (*g* = 0.742; 95% CI 0.372–1.112; *p* < 0.001; *I* ^2^ = 83%) (Figure 3B), and the KYN/TRP ratio (*g* = 0.656; 95% CI 0.423–0.949; *p* < 0.001; *I* ^2^ = 84%) (Figure 4A) increased. In contrast, the QA/KYNA ratio did not change significantly from pre to post exercise (*g* = 0.023; 95% CI −0.639 to 0.684; *p* = 0.94; *I* ^2^ = 94%) (Figure 4B).

During the recovery phase, TRP remained significantly reduced (*g* = −0.767; 95% CI −1.025 to −0.510; *p* < 0.001; *I* ^2^ = 75%) (Figure S3A), whereas KYNA (*g* = 0.810; 95% CI 0.281–1.339; *p* = 0.003; *I* ^2^ = 92%) (Figure S3D), and the KYN/TRP ratio (*g* = 0.372; 95% CI 0.099–0.645; *p* = 0.007; *I* ^2^ = 79%) (Figure S3E) remained elevated. In contrast, elevations in KYN (*g* = −0.203; 95% CI −0.495 to 0.090; *p* = 0.17; *I* ^2^ = 82%) (Figure S3B) and QA (*g* = 0.144; 95% CI −0.002 to 0.290; *p* = 0.05; *I* ^2^ = 0%) (Figure S3C) did not remain, with KYN tending to return toward baseline and the immediate QA response being attenuated. The QA/KYNA ratio remained unchanged (*g* = 0.099; 95% CI −0.319 to 0.518; *p* = 0.64; *I* ^2^ = 86%) (Figure S3F).

Across both temporal windows, high heterogeneity was observed for most outcomes, indicating that a substantial proportion of the observed variability reflected true differences in effect sizes rather than sampling error alone. Because heterogeneity exceeded the commonly accepted threshold of 75% for most analyses, prespecified subgroup analyses were conducted to explore potential sources of between‐study variability, including health status, training status, and exercise modality.

### 3.4 Subgroup Analyses

Table 3 summarizes the results of the subgroup analyses exploring potential moderators of the acute KYN pathway response to exercise. Significant differences between subgroups were observed for TRP and KYNA. Healthy individuals exhibited larger reductions in TRP than clinical populations (*g* = −0.526 vs. −0.311; Q‐between = 3.85, *p* = 0.05) and greater increases in KYNA (*g* = 0.713 vs. 0.171; Q‐between = 15.00, *p* < 0.001). Similarly, trained individuals showed larger KYNA responses than untrained individuals (*g* = 0.688 vs. 0.010; Q‐between = 26.26, *p* < 0.001).

**TABLE 3:** Subgroup analysis of the acute effect of exercise on tryptophan and kynurenine pathway metabolites across the immediate and recovery assessment windows.

	Interventions	Meta‐analysis		Heterogeneity	
		Hedge's g (IC 95%)	*p*	*Q*	*p*
**TRP**					
**Sample**					
Healthy	19	−0.526 (−0.645, −0.408)	< 0.001	3.85	0.05
Clinical condition	6	−0.311 (−0.490, −0.132)	0.001		
**Training status**					
Trained	11	−0.430 (−0.590, −0.271)	< 0.001	4.28	0.11
Untrained	5	−0.334 (−0.524, −0.145)	0.001		
Unreported	9	−0.595 (−0.764, −0.426)	< 0.001		
**Exercise modality**					
Aerobic	20	−0.509 (−0.616, −0.402)	< 0.001	5.26	0.02
Resistance	5	−0.184 (−0.440, 0.072)	0.160		
**KYN**					
**Sample**					
Healthy	17	0.147 (0.031, 0.262)	0.01	0.37	0.54
Clinical condition	7	0.087 (−0.067, 0.241)	0.27		
**Training status**					
Trained	10	0.199 (0.039, 0.359)	0.01	4.69	0.09
Untrained	5	−0.048 (−0.231, 0.134)	0.60		
Unreported	9	0.173 (0.029, 0.318)	0.01		
**Exercise modality**					
Aerobic	19	0.116 (0.015, 0.216)	0.02	0.22	0.64
Resistance	5	0.176 (−0.057, 0.409)	0.13		
**QA**					
**Sample**					
Healthy	8	0.401 (0.228, 0.573)	< 0.001	3.53	0.06
Clinical condition	3	0.129 (−0.096, 0.354)	0.26		
**Training status**					
Trained	5	0.374 (0.130, 0.618)	0.003	3.62	0.16
Untrained	3	0.129 (−0.096, 0.354)	0.26		
Unreported	3	0.427 (0.183, 0.672)	0.001		
**Exercise modality**					
Aerobic	8	0.301 (0.144, 0.458)	< 0.001	0.001	0.98
Resistance	3	0.297 (0.019, 0.575)	0.03		
**KYNA**					
**Sample**					
Healthy	10	0.713 (0.518, 0.908)	< 0.001	15.00	< 0.001
Clinical condition	5	0.171 (−0.023, 0.364)	0.08		
**Training status**					
Trained	7	0.688 (0.436, 0.940)	< 0.001	26.26	< 0.001
Untrained	3	0.010 (−0.205, 0.224)	0.93		
Unreported	5	0.784 (0.532, 1.036)	< 0.001		
**Exercise modality**					
Aerobic	12	0.467 (0.318, 0.616)	< 0.001	0.87	0.35
Resistance	3	0.284 (−0.070, 0.638)	0.12		

Abbreviations: KYN, kynurenine; KYNA, kynurenic acid; QA, quinolinic acid; TRP, tryptophan.

Exercise modality also influenced TRP responses, with aerobic exercise producing greater reductions than resistance exercise (*g* = −0.509 vs. −0.184; Q‐between = 5.26, *p* = 0.02). No significant subgroup differences were detected for KYN or QA according to health and training status, or exercise modality (all *p* > 0.05).

Although no additional between‐subgroup differences reached statistical significance, healthy and trained participants consistently showed larger effect sizes, whereas no significant exercise‐induced changes in KYN, QA, or KYNA were detected within clinical or untrained populations.

### 3.5 Sensitivity Analyses and Publication Bias

Sensitivity analyses excluding influential studies did not materially change the magnitude, direction, or significance of the pooled estimates. Funnel plots for the primary outcomes are shown in Data S4 and revealed no clear evidence of publication bias. However, these findings should be interpreted cautiously for outcomes based on a small number of studies, as funnel plot reliability is limited in smaller meta‐analyses.

## 4 Discussion

### 4.1 General Findings

The main finding of this systematic review and meta‐analysis is that a single exercise session elicits a transient kynurenine metabolic signature across human trials. This response is characterized by reductions in circulating TRP and increases in the KYN/TRP ratio, accompanied by immediate elevations in KYN and the downstream metabolites QA and KYNA. While KYN and QA returned toward baseline during recovery, reductions in TRP and elevations in KYNA and the KYN/TRP ratio persisted, indicating distinct temporal patterns within the pathway. Interestingly, the QA/KYNA ratio remained unchanged in both time windows despite changes in QA and KYNA. Qualitative evidence further indicated increases in PIC [29, 47] and in xanthurenic acid [29], whereas anthranilic acid responses were inconsistent across studies [27, 28].

The magnitude of the exercise‐induced KYN pathway responses was influenced by health and training status, as well as exercise modality. Healthy individuals exhibited greater changes in TRP and KYNA than clinical populations, whereas trained individuals showed larger KYNA responses than untrained individuals. Aerobic exercise was associated with greater TRP reductions than resistance exercise. However, because most available studies investigated aerobic exercise, conclusions regarding modality‐specific effects should be interpreted cautiously.

### 4.2 Population‐Specific Responsiveness

Baseline health status appears to influence KYN pathway responsiveness. Although both healthy and clinical populations exhibited reductions in TRP following exercise, healthy individuals showed greater TRP reductions and larger increases in KYNA than clinical populations. However, these findings should be interpreted cautiously, given the limited evidence and heterogeneous clinical populations studied. Lower exercise volumes, workloads, and muscle mass recruitment may also have contributed to the observed differences.

This blunted responsiveness may reflect chronic inflammation, altered baseline TRP metabolism, impaired immune signaling, mitochondrial dysfunction, and reduced metabolic flexibility, all of which can affect substrate availability and enzymatic regulation during acute stress [15, 17].

Disease‐specific findings further suggest that clinical phenotype influences pathway behavior. In multiple sclerosis, acute exercise responses varied according to phenotype and exercise intensity, with relapsing–remitting patients generally showing greater responsiveness than patients with secondary progressive disease, which may be due to greater disability typically observed in the latter phenotype [48, 51]. In patients with bipolar personality disorder, acute exercise increased KYNA and KYNA‐derived ratios alongside an interleukin‐6 response, suggesting a beneficial shift in the KYN pathway [52].

In patients with type 2 diabetes, individuals retained functional responsiveness of the KYN‐to‐KYNA conversion pathway during intense exercise, as indicated by exercise‐induced increases in KYNA. This observation was accompanied by increased peroxisome proliferator‐activated receptor gamma coactivator 1‐alpha 1 (PGC‐1α1) gene expression in skeletal muscle, suggesting preserved adaptive metabolic signaling despite baseline metabolic dysfunction [34]. In contrast, patients with heart failure showed altered downstream metabolite handling, including delayed QA accumulation during recovery [53]. In prostate cancer survivors, a single cycling session reduced TRP and increased the KYN/TRP ratio without altering downstream KYN metabolites [50]. Overall, evidence in clinical populations remains limited, with most observations derived from single studies.

Taken together, these findings highlight the importance of baseline inflammatory and metabolic status in shaping exercise‐induced KYN pathway responses, while also highlighting the need for additional disease‐specific investigations in clinical populations.

### 4.3 Mechanistic and Clinical Interpretation

A single exercise session appears to modulate the KYN pathway through integrated metabolic and immune responses [20]. Exercise‐induced increases in cytokines, myokines, catecholamines, and immune cell mobilization likely contribute to enhanced TRP degradation and downstream metabolite production [24, 54], suggesting increased pathway activity and metabolite turnover rather than sustained accumulation of circulating intermediates [55, 56, 57, 58].

This interpretation is also supported by mechanistic evidence from both human and animal studies demonstrating transient exercise‐induced regulation of key enzymes involved in KYN metabolism, including IDO and KAT isoforms [19, 21, 33, 34, 44, 59].

Similarly, human studies included in the present review reported acute alterations in the peripheral blood mononuclear cell (PBMC) expression of IDO1, IDO2, and KAT isoforms following aerobic exercise, supporting the concept that exercise induces coordinated systemic and tissue‐level regulation of the KYN pathway [24, 44].

### 4.4 Acute Molecular Signaling and Adaptive Implications

These acute responses may also have broader clinical relevance. Given the involvement of KYN metabolites in inflammation, oxidative metabolism, neurobiology, and immune regulation, these acute alterations may represent biologically relevant mechanisms contributing to exercise adaptation and systemic interorgan communication [15, 17, 60].

Although the metabolic effects of a single exercise session are transient, repeated exposure to these perturbations may contribute to long‐term adaptations in KYN pathway regulation [15]. Skeletal muscle signaling pathways appear to play a central role in this process. Activation of PGC‐1α has been associated with increased expression of KATs, favoring the peripheral conversion of KYN into KYNA and shifting metabolite balance toward potentially neuroprotective and anti‐inflammatory pathways [21, 61]. In fact, acute cycling exercise increased skeletal muscle PGC‐1α1 mRNA expression together with circulating KYNA and the KYNA/KYN ratio in both healthy individuals and patients with type 2 diabetes [34].

Exercise also activates transcriptional and immunometabolic machinery regulating KYN metabolism in circulating immune cells. Endurance exercise increased KAT4 gene expression in PBMCs alongside elevations in KYNA and KYNA/KYN ratio [24], while subsequent work demonstrated acute increases in IDO1, IDO2, KAT3, and KAT4 gene expression together with activation of aryl hydrocarbon receptor (AhR)‐related transcriptional targets following both moderate‐ and high‐intensity exercise [44]. However, transcript‐level responses should not be interpreted as direct evidence of corresponding protein‐level changes, as protein measurements were limited and did not consistently parallel gene expression responses.

Beyond transcriptional regulation, KYN pathway metabolites may also exert important signaling functions through activation of AhR‐dependent pathways involved in immune cell differentiation, inflammatory signaling, mitochondrial function, and metabolic regulation. In this context, activation of the KYN–AhR axis has been associated with modulation of regulatory T‐cell (T_reg_) differentiation and broader immune tolerance mechanisms, suggesting that acute exercise‐induced alterations in KYN pathway metabolism may contribute to acute immunoregulatory adaptations with potential implications for longer‐term immune regulation through coordinated cytokine and immune signaling responses during exercise and recovery [21, 62, 63].

Experimental evidence further indicates that exercise‐induced activation of skeletal muscle PGC‐1α1 signaling enhances KAT expression and integrates KYN metabolism with mitochondrial bioenergetics, oxidative metabolism, fatigue resistance, and the malate–aspartate shuttle [21]. Importantly, the KYN pathway is also the only de novo route for NAD^+^ synthesis, linking exercise‐induced alterations in TRP metabolism to cellular energy homeostasis and immune cell function [2, 8, 62, 64]. The transient increase in QA observed immediately after exercise is consistent with this concept, although evidence linking acute exercise‐induced changes in QA to enhanced NAD^+^ synthesis remains limited.

Nevertheless, recent findings by Walzik et al. (2025) [62] demonstrated acute exercise‐induced increases in the expression of NAD^+^‐related enzymes, including nicotinamide phosphoribosyltransferase (NAMPT) and quinolinate phosphoribosyltransferase (QPRT), in PBMCs, supporting the notion that exercise‐induced modulation of the KYN pathway may contribute to immunometabolic adaptation across multiple tissues and cell types.

However, molecular responsiveness is unlikely to be universal across tissues or biological compartments. Supporting this concept, Isung et al. [43] reported distinct responses between plasma and cerebrospinal fluid following acute exercise, suggesting compartment‐specific regulation of KYN metabolism. Similarly, Jonsson et al. (2022) [33] observed reductions in circulating TRP and KYN despite unchanged skeletal muscle KAT4 expression, suggesting that tissue‐specific regulation, in addition to sampling timing and exercise modality, may substantially influence the molecular signature of the acute response. This limited concordant multi‐level evidence highlights the need for future studies concurrently integrating circulating metabolite measurements with tissue‐specific analyses of gene expression and protein abundance within the same participants and sampling windows to better characterize the mechanisms linking acute exercise‐induced metabolic signaling to adaptive regulation of the KYN pathway.

In parallel, microbiota‐derived indoles may also contribute to exercise‐induced immunometabolic signaling through gut–immune interactions and AhR activation [65, 66, 67]. Although these metabolites were beyond the scope of the present review, emerging evidence suggests they may represent an additional pathway linking exercise, host metabolism, and immune regulation [68].

### 4.5 Strengths and Limitations

This study provides the first quantitative synthesis of the acute effects of exercise on KYN pathway metabolism in humans, integrating evidence across different populations, exercise protocols, and sampling strategies. Importantly, the meta‐analytic approach enabled the quantification of effect sizes, providing estimates of the magnitude of acute exercise‐induced changes in KYN pathway metabolites. Subgrouping according to health and training status, as well as exercise modality, further allowed for the identification of important moderators influencing the metabolic response.

Some limitations should be considered. Substantial heterogeneity across studies, including differences in participant characteristics, exercise protocols, and sampling timing, reduced the precision of pooled estimates. Many studies had small sample sizes and did not consistently control for factors known to influence TRP metabolism, such as diet, circadian variation, medication or supplement intake, and baseline inflammatory or metabolic status. In addition, the limited number of studies in specific clinical subgroups restricted stronger conclusions regarding disease‐specific responses. The limited availability of matched metabolite, gene‐expression, and protein measurements also precluded quantitative integration across molecular levels. Finally, publication bias analyses were limited for outcomes presented by a small number of studies.

## 5 Conclusion

This systematic review and meta‐analysis provide evidence that exercise acutely modulates the KYN pathway metabolism in humans. Exercise induced a time‐dependent KYN pathway metabolic response characterized by sustained reductions in TRP in combination with elevations in KYNA and in the KYN/TRP ratio (up to 3 h), and short‐lived (up to 5 min) increases in KYN and QA immediately after exercise. Some of these responses were strongly influenced by baseline health and training status as well as exercise modality, with healthy individuals, trained status, and aerobic exercise showing the most robust effects.

The findings further suggest that a single exercise session acts as a transient but biologically relevant metabolic stimulus capable of modifying KYN pathway partitioning. Repeated exposure to these acute perturbations may contribute to longer‐term adaptations in TRP metabolism and immunometabolic regulation. Future studies using standardized exercise protocols, precise temporal sampling, and integrated molecular approaches are needed to clarify the mechanisms underlying these responses and determine their relevance for chronic adaptation and clinical outcomes.

## Author Contributions

**Marcus Vinicius dos Santos Rangel:** conceptualization, methodology, data curation, investigation, formal analysis, writing – original draft preparation, writing – review and editing. **Gabriela Andrade Paz:** methodology, writing – review and editing. **Maria das Graças C. Souza:** methodology, writing – review and editing. **Marie Kupjetz:** methodology, writing – review and editing. **Tiffany Y. Wences Chirino:** methodology, writing – review and editing. **Philipp Zimmer:** methodology, supervision, writing – review and editing. **Paulo Farinatti:** methodology, supervision, writing – review and editing. **Juliana Pereira Borges:** conceptualization, methodology, data curation, investigation, formal analysis, supervision, writing – original draft preparation, writing – review and editing. All authors have read and agreed to the published version of the manuscript.

## Funding

This work was supported by the Conselho Nacional de Desenvolvimento Científico e Tecnológico (CNPq) under grants [number 404204/2023‐6 and 305332/2024‐4], the Fundação Carlos Chagas Filho de Amparo à Pesquisa do Estado do Rio de Janeiro (FAPERJ) under grant [number E‐26/204.202/2025], and the Fundação Carlos Chagas Filho de Amparo à Pesquisa do Estado do Rio de Janeiro (FAPERJ) under grant [number E‐26/210.552/2026].

## Conflicts of Interest

The authors declare no conflicts of interest.

## Supporting information


**Data S1:** Search strategy.

**Data S2:** Risk of bias assessment of included studies using the ROBINS‐I tool. Traffic‐light plot summarizing domain‐level and overall risk of bias for each included study. The seven domains assessed were: D1, bias due to confounding; D2, bias due to selection of participants; D3, bias in classification of interventions; D4, bias due to deviations from intended interventions; D5, bias due to missing data; D6, bias in measurement of outcomes; and D7, bias in selection of the reported result. Symbols represent the level of risk of bias: green (+), low risk; yellow (−), moderate risk; red (×), serious risk; dark red (!), critical risk. The final column indicates the overall risk of bias judgment for each study.

**Data S3:** Hedges' g pooled effect sizes and 95% confidence intervals for the recovery responses (> 5 min up to 3 h after exercise) on TRP (Panel A, *k* = 10, 16 interventions) and KYN (Panel B, *k* = 9, 14 interventions). Numerals between brackets represent different interventions within the same study.

**Data S4:** Funnel plot of precision for the immediate post‐exercise (≤ 5 min after exercise cessation) and recovery (> 5 min to 3 h post‐exercise) analyses of changes in tryptophan after exercise. Blue diamond: observed g; red diamond: adjusted g. Three missing trials were detected.

## Data Availability

No new datasets were generated during this study. All data analyzed are available within the published articles included in this systematic review and meta‐analysis and in the Supporting Information provided with this article.
