# Supplementary material for: A Single Exercise Session Elicits a Transient Kynurenine Metabolic Signature in Health and Disease: A Systematic Review and Meta‐Analysis
Source: FASEB J. 2026 Oct 13;40(20):e72349. doi: 10.1096/fj.202603422R (PMC13660704; doi:10.1096/fj.202603422R)
Supplement: Supplementary file 1 [file FSB2-40-e72349-s001.docx]

Supplemental Material 2

Risk of bias assessment of included studies using the ROBINS-I tool.
Traffic-light plot summarizing domain-level and overall risk of bias for each included study. The seven domains assessed were: D1, bias due to confounding; D2, bias due to selection of participants; D3, bias in classification of interventions; D4, bias due to deviations from intended interventions; D5, bias due to missing data; D6, bias in measurement of outcomes; and D7, bias in selection of the reported result. Symbols represent the level of risk of bias: green (+), low risk; yellow (–), moderate risk; red (×), serious risk; dark red (!), critical risk. The final column indicates the overall risk of bias judgment for each study.
