# Supplementary material for: A Single Exercise Session Elicits a Transient Kynurenine Metabolic Signature in Health and Disease: A Systematic Review and Meta‐Analysis
Source: FASEB J. 2026 Oct 13;40(20):e72349. doi: 10.1096/fj.202603422R (PMC13660704; doi:10.1096/fj.202603422R)
Supplement: Supplementary file 2 [file FSB2-40-e72349-s002.docx]

Supplemental Material 1

Search Strategy

("exercise" OR "exercises" OR “concurrent exercise” OR walking OR bicycling[mesh] OR bicycling OR bicycle OR cycling OR "cycle ergometer" OR "arm ergometer" OR running[mesh] OR treadmill* OR "endurance training" OR "aerobic endurance" OR "weight lifting" OR "weight training" OR "resistance training" OR "strength training" OR "isometric training" OR "hand grip" OR "training duration" OR "training frequency" OR "training intensity" OR "anaerobic training") AND (tryptophan OR kynurenine OR “kynurenic acid” OR quinolinic OR kynurenic OR “indoleamine 2, 3-dioxygenase” OR “Tryptophan-2,3-dioxygenase” OR “kynurenine aminotransferases” OR “3-hydroxykynurenine” OR “aryl hydrocarbon receptor” OR “picolinic acid” OR anthranilic) NOT ("Cross-Sectional Studies"[mesh] OR "Case Reports"[pt] OR Comment[pt] OR Editorial[pt] OR Letter[pt] OR Review[pt] OR "case control"[tiab] OR "case report"[tiab] OR "case study"[tiab] OR guidelines[ti] OR "inspiratory muscle-training"[tiab] OR "manipulation, osteopathic"[MeSH] OR "osteopathic manipulation"[tiab] OR “Cross-Sectional Study"[ti] OR "cohort study" OR "Case Report" OR Comment OR Editorial OR Letter OR "Keynote lecture" OR Review OR "meta-analysis" OR "meta-analysis" OR "factor analysis" OR "principal component analysis" OR "correlation study" OR "correlates" OR "case control" OR "case study" OR "epidemiologic study" OR "position stand)
