# Supplementary material for: A Single Exercise Session Elicits a Transient Kynurenine Metabolic Signature in Health and Disease: A Systematic Review and Meta‐Analysis
Source: FASEB J. 2026 Oct 13;40(20):e72349. doi: 10.1096/fj.202603422R (PMC13660704; doi:10.1096/fj.202603422R)
Supplement: Supplementary file 3 [file FSB2-40-e72349-s003.docx]

Supplemental Material 4

Funnel plot of precision for the immediate post-exercise (≤ 5 min after exercise cessation) and recovery (> 5 min to 3 h post-exercise) analyses of changes in tryptophan after exercise. Blue diamond: observed g; red diamond: adjusted g. Three missing trials were detected.

Funnel plot of precision for the immediate post-exercise (≤ 5 min after exercise cessation) and recovery (> 5 min to 3 h post-exercise) analyses of changes in kynurenine after exercise. Blue diamond: observed g; red diamond: adjusted g. No missing trials were detected.

Funnel plot of precision for the immediate post-exercise (≤ 5 min after exercise cessation) and recovery (> 5 min to 3 h post-exercise) analyses of changes in quinolinic acid after exercise. Blue diamond: observed g; red diamond: adjusted g. No missing trials were detected.

Funnel plot of precision for the immediate post-exercise (≤ 5 min after exercise cessation) and recovery (> 5 min to 3 h post-exercise) analyses of changes in kynurenic acid after exercise. Blue diamond: observed g; red diamond: adjusted g. One missing trial was detected.

Funnel plot of precision for the immediate post-exercise (≤ 5 min after exercise cessation) and recovery (> 5 min to 3 h post-exercise) analyses of changes in kynurenine-tryptophan ratio after exercise. Blue diamond: observed g; red diamond: adjusted g. No missing trials were detected.

Funnel plot of precision for the immediate post-exercise (≤ 5 min after exercise cessation) and recovery (> 5 min to 3 h post-exercise) analyses of changes in quinolic acid-kynurenic acid ratio after exercise. Blue diamond: observed g; red diamond: adjusted g. No missing trials were detected.
