# Supplementary material for: A Single Exercise Session Elicits a Transient Kynurenine Metabolic Signature in Health and Disease: A Systematic Review and Meta‐Analysis
Source: FASEB J. 2026 Oct 13;40(20):e72349. doi: 10.1096/fj.202603422R (PMC13660704; doi:10.1096/fj.202603422R)
Supplement: Supplementary file 4 [file FSB2-40-e72349-s004.docx]

Supplemental Material 3

A

B

Hedges’ g pooled effect sizes and 95% confidence intervals for the recovery responses (> 5 min up to 3 hours after exercise) on TRP (Panel A, k = 10, 16 interventions) and KYN (Panel B, k = 9, 14 interventions). Numerals between brackets represent different interventions within the same study.

C

D

Hedges’ g pooled effect sizes and 95% confidence intervals for the recovery responses (> 5 min up to 3 hours after exercise) on QA (Panel A, k = 6, 8 interventions) and KYNA (Panel B, k = 9, 16 interventions). Numerals between brackets represent different interventions within the same study.

F

E

Hedges’ g combined effect sizes and 95% confidence intervals for the recovery responses (> 5 min up to 3 hours after exercise) on KYN/TRP (Panel A, k = 9, 12 interventions) and QA/KYNA (Panel B, k = 5, 8 interventions). Numerals between brackets represent different interventions within the same study.
